# Weight Management Apps in Saudi Arabia: Evaluation of Features and Quality

**DOI:** 10.2196/19844

**Published:** 2020-10-26

**Authors:** Dalal M Alshathri, Abeer S Alhumaimeedy, Ghada Al-Hudhud, Aseel Alsaleh, Sara Al-Musharaf, Ghadeer S Aljuraiban

**Affiliations:** 1 Department of Nutrition Security Forces Hospital Program Riyadh Saudi Arabia; 2 Department of Software Engineering College of Computer and Information Sciences King Saud University Riyadh Saudi Arabia; 3 Department of Information Technology College of Computer and Information Sciences King Saud University Riyadh Saudi Arabia; 4 Family and Community Medicine Department College of Medicine Arabian Gulf University Manama Bahrain; 5 Department of Community Health Sciences College of Applied Medical Sciences King Saud University Riyadh Saudi Arabia

**Keywords:** mHealth, eHealth, smartphone, obesity, weight management, mobile apps, MARS, six sigma

## Abstract

**Background:**

Weight management apps may provide support and management options for individuals with overweight and obesity. Research on the quality of weight management mHealth apps among the Saudi population is insufficient despite frequent use.

**Objective:**

The aims of this study were to explore user perceptions of weight management apps, explore reasons for starting and stopping app use, appraise the quality of weight management apps available in the App Store, and compare the features currently available within the app market and those that are most desirable to weight management app users.

**Methods:**

A web-based survey consisted of 31 open and closed questions about sociodemographic information, general health questions, app use, app user perceptions, and discontinuation of app use. The quality of the weight management apps available on the App Store was assessed using the Mobile App Rating Scale and evidence-based strategies. We also used six sigma evaluations to ensure that the quality measured by the tools consistently meets customer expectations.

**Results:**

Data from the survey were analyzed. Of the respondents, 30.17% (324/1074) had used a weight management app, 18.16% (195/1074) used the apps and stopped, and 51.68% (555/1074) had never used a weight management app. Of apps mentioned, 23 met the inclusion criteria. The overall average Mobile App Rating Scale quality of apps was acceptable; 30% (7/23) received a quality mean score of 4 or higher (out of 5), and 30% (7/23) did not meet the acceptability score of 3 or higher. Evidence-based strategy results showed that feedback was not observed in any of the apps, and motivation strategy was observed in only 1 app. The sigma results of evidence-based strategies reflect that most of the apps fail to pass the mean.

**Conclusions:**

App users desired a feature that allows them to communicate with a specialist, which is a missing in the available free apps. Despite the large number and accessibility of weight management apps, the quality and features of most are variable. It can be concluded from six sigma results that passing the mean does not ensure that the quality is consistently distributed through all app quality properties and Mobile App Rating Scale and evidence-based strategies do not give developers an indication of the acceptance of their apps by mobile users. This finding stresses the importance of reevaluating the passing criterion, which is ≥50% for designing an effective app.

## Introduction

Obesity, a multifactorial health problem related to behavioral factors such as physical activity and diet [1], is a major public health concern worldwide, and its incidence nearly doubled from 1975 to 2016 according to a long-term analysis of trends using BMI [2]. In 2013, the Saudi Health Interview Survey reported that the prevalence of obesity was 29%, higher in women than men (33.5% vs 24.1%), and expected to continue to increase [3].

One of the factors causing individuals to have a more sedentary lifestyle is the use of smartphones [4]. In 2016, the percentage of Saudi people using smartphones reached 88% [5], and in 2018, the penetration rate of mobile cellular subscriptions was approximately 129% of the population according to the Communication and Information Technology Commission [6]. Data from large-scale surveys have showed that weight management apps were some of the most popular among medical and public health apps (mHealth apps) [7-9]. The use of these weight management apps showed effectiveness according to a systematic review and meta-analysis of 12 clinical trials that reported significant weight losses of 1 kg relative to traditional weight reduction interventions or intensive consulting [10].

A recent survey in Saudi Arabia aimed to explore weight management app use, barriers to use, and reasons for discontinued use among smartphone users [11]. The study demonstrated that more than 40% of participants used weight management apps and more than half of the app users were overweight or obese. However, a limited number of studies have assessed the quality of weight management apps using clear, identifiable, and justifiable quality assessment measures [7].

The quality evaluation of apps plays a vital role in assisting with the development and improvement of mHealth apps. Recently, several software product measures and metrics have been used to evaluate app quality, but many of these metrics are technical and highly dependent on the software type [12]. The Mobile App Rating Scale (MARS) is one of many tools for evaluating mHealth apps in smartphones [13], and it has been used in different studies to evaluate a variety of mHealth apps [14-16]. Apps can also be evaluated for their level of adherence to evidence-based strategies (EBS) by characterizing them depending on the presence or absence of app features [17]. Additionally, six sigma is a unique data-driven process used to test and analyze the policies, procedures, and measures of a quality plan to help software engineers easily detect quality discrepancies in apps [18]. In software engineering, six sigma is used to evaluate and control software quality such as that for mobile weight management apps [18].

Given the limited available studies that evaluate the quality of popular weight management apps, the aim of this study was to identify app users’ perceptions and reasons for starting and stopping app use and evaluate the quality of weight management apps using two measurement tools: MARS and EBS. We also used six sigma methodology to ensure that the quality measured by the tools consistently meets customer expectations and determine whether the quality of the app is related to app use among users in Saudi Arabia.

## Methods

### Design and Sample

This cross-sectional web-based survey was conducted with Saudi smartphone users. Over 4 months, smartphone users were invited to participate in an anonymous, web-based survey hosted on a Microsoft platform. The survey link was advertised through social media and university portals. To increase the number of participants and their diversity, Twitter ads were used to promote the survey link in 8 Saudi cities, including Baha, Eastern, Tabuk, Asir, Makkah, Almadeinah, Hail, and Jazan. The ad ran during the second week of September 2019 for 7 days. The survey was open to anyone who wanted to participate, and both app users and nonusers were invited. Survey responses were collected over a 4-month period. The King Saud University institutional review board approved the study (reference E-19-4001) in May 2019. No personal identifying information was collected, and participants had the right to refuse to participate in the study. Informed consent was a requirement for participation. The online survey was in accordance with the Checklist for Reporting Results of Internet e-Surveys (CHERRIES, Multimedia Appendix 1) [19].

Participants aged younger than 18 years, those with inconsistent or illogical responses (ie, participant reported a weight of –10 kg), and those who were app users and failed to answer more than 50% of the questions on app use were excluded.

The sample size was calculated based on Saudi Arabia’s current population of 10.7 million adults aged 18 years and older [20] and that 88% of adult Saudis were smartphone users at the time of the research [5]. A confidence level of 95% and a precision of 5% were used. Given these parameters, the minimum sample required for the analyses to have a power of 95% was 385 individuals.

### Survey Items

The survey consisted of 31 questions encompassing the following domains: (1) sociodemographic characteristics (age, sex, residency, nationality, income, education, and employment), (2) general health (tobacco use, weight, height, medical diagnoses, physical activity, and diet), (3) app use (app name, reason for download, use pattern, app features, and whether app was recommended by health care provider), (4) app user perceptions (effectiveness, security, and accuracy), and (5) reason for discontinuing use of the app. These questions were obtained from a questionnaire used in the United States (Multimedia Appendix 2) [9].

The questionnaire was tested on a small sample of app users and nonusers (n=10). Questions and question order were revised based on feedback and results from the test to reduce response bias and enhance response time.

The final web-based questionnaire was presented in 3 steps. The first screen asked participants for their informed consent. Consent was a required response before the respondent could advance to the next screen. The second screen contained sociodemographic and general health questions. The last screen contained questions on app use, stop use, and user perceptions. The back icon on each screen allowed participants to edit previous answers. The survey comprised a mix of open and closed questions and took between 5 and 9 minutes to complete.

### App Search Strategy

In Saudi Arabia, the use of iOS devices increased by 3.6% and Android decreased by 3% from June to October 2019 [21]. Therefore, our search was limited to iOS users (Apple App Store). The App Store was searched twice for apps using English and Arabic search terms (weight loss, diet, and weight management). Through the search strategy, we aimed to find a large proportion of apps within our designated time frame. Thus, we used 6 search terms (3 in English and 3 in Arabic) with an arbitrary decision to include the first 10 displayed apps from each search. The search was done in November 2019 using a newly created Apple ID without any search history. The search was not filtered by any attribute as this feature does not exist in the App Store (iOS 13). The first 10 displayed apps from each search were reviewed based on predefined inclusion criteria: free, language is English or Arabic, made for the average consumer, and related to weight loss.

### Quality Assessment Tools for Weight Management Apps

#### Mobile App Rating Scale

The quality of the weight management apps was assessed independently by two investigators (DA and GA) using MARS; any discrepancy was reviewed by a third investigator (ASA) and resolved by consensus. MARS contains 23 items rated on a 5-point scale (1.0=inadequate, 2.0=poor, 3.0=acceptable, 4.0=good, and 5.0=excellent) [13]. A total of 19 questions formed the objective quality section, which was divided into 4 scales: engagement, functionality, aesthetics, and information quality. Four questions formed the subjective quality section that evaluated user satisfaction. Apps were evaluated on an iPhone (iOS 13), and their star ratings from the App Store were obtained for further analysis. For the subjective scale, an average rating was taken.

#### Evidence-Based Strategy Assessment

App evidence-based strategies were characterized depending on the presence or absence of app features [17]. Strategies and indicators of adherence were as follows:

- Presence of self-monitoring capabilities for weight, meals, nutrition (including protein, fats, carbohydrates, fiber, and water), and physical activity
- Presence of goal setting with or without customization
- Healthy eating support including information, education, and skills development
- Physical activity support including information, education, and skills development
- Social support such as online communication with other users
- Weight and/or health assessment with or without personalization
- Motivational strategies including prompts, rewards, or a gamified design
- Personalized feedback

Apps were independently reviewed for their level of adherence to EBS by two investigators (DA and GA) with discrepancies reviewed by a third investigator (ASA) and resolved by consensus.

#### Six Sigma Evaluations

In software engineering, six sigma is used to evaluate and control software quality [22]. Software engineers use six sigma statistical methods like run, control charts, and process capability index (Cpk) to examine the software quality based on the quality measurements and quality standards. Six sigma is a data-driven, problem-solving process that consists of 5 stages, the Define-Measure-Analyze-Improve-Control process, to achieve six sigma goals. The results are then analyzed within the context of the software, and improvements are suggested based on the analysis outcomes (ie, if the sigma level is less than 6) [22].

Two researchers (ASA and GH) independently applied six sigma to evaluate apps quality based on quality data of MARS. Results were compared with MARS means, and the relation of the quality of the app to app use was identified.

Six sigma evaluation measures the quality of apps based on their behavior in all defined quality attributes, and based on this behavior it predicts app behavior on an undefined one. More specifically, we used the Cpk of six sigma to evaluate how close the app quality is to customer expectations considering its natural variability [22]. Cpk is a statistical measure of a software quality (ie, the ability of the software to meet software quality standard measures). The Cpk measures the natural variation of software quality relative to the quality standards limits. In addition, it allows the comparison of different software with respect to how well software meets quality standard limits. In a relative manner and within the context of this investigation, we used the limits of MARS (5 is considered excellent; 3 is considered acceptable) as the quality standard limits [13]. Thus, if a certain app scores high in six sigma, then that app will continually meet quality attribute limits of MARS and is expected to meet the limits of other quality attributes when considered [22]. The larger the Cpk, the higher the app quality. To calculate the Cpk, we used the following equation:

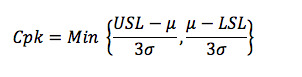



Where USL is the upper limit of customer expectations, LSL is the lower limit of the customer expectations, µ is the data mean, and σ is the standard deviation of the sample data.

Based on the Cpk, we can determine the sigma level of the app according to the following specification [22]:

- Cpk 0.33: sigma level 1
- Cpk 0.67: sigma level 2
- Cpk 1: sigma level 3
- Cpk 1.33: sigma level 4
- Cpk 1.67: sigma level 5
- Cpk 2: sigma level 6
- Cpk <0: limit is irrelevant

Levels below 3 are considered poor quality, and levels 3 and above are considered good quality. Level 6 represents the best quality [22].

### Statistical Analysis

Data analysis was performed using SPSS Statistics version 24.0 (IBM Corporation). Numerical variables are represented as means and standard deviations, and categorical variables are represented as percentages. BMI was calculated as the weight in kilograms divided by the square of the height in meters (kg/m²). The BMI categories were classified as follows: underweight (BMI<18.5), normal weight (18.5≤BMI≤24.9), overweight (25.0≤BMI≤29.9), moderate obesity (30.0≤BMI≤34.9), and severe obesity (BMI≥35).

Participants were stratified based on the following app use categories: (1) users (participants reported that they use a weight management app), (2) ex-users (participants reported that they used weight management apps and then stopped), and (3) nonusers (participants reported that they never used a weight management app). Analysis was done by user category with the data on app use and app user perceptions. Simple logistic regression analysis was applied to identify the association between BMI and use of a weight management app. Linear regression analysis was used to determine the relationship between the ranking of the apps in the App Store with the MARS score and also between the MARS score and the EBS criteria.

## Results

### Sociodemographic Characteristics and Health Status

The results of the sociodemographic characteristics for all participants stratified by use pattern (user, ex-user, or nonuser) are presented in Table 1. A total of 1209 people responded to the survey. Of the participants who read the welcome page and proceeded to consent, 98.68% (1193/1209) agreed to participate in the survey. The data were excluded in the analysis if the respondents were non-Saudi, aged younger than 18 years, provided inconsistent or illogical answers (ie, participant reported his weight as –10 kg), or if the participants are app users and did not answer more than 50% of questions on app use, which left 1074 responses for further analysis.

Of the respondents, 30.17% (324/1074) used a weight management app, 18.16% (195/1074) used an app and stopped, and 51.68% (555/1074) had never used a weight management app. The majority of the respondents were aged 18 to 31 years (785/1074, 73.09%); 69.93% (751/1074) were female and 30.07% (323/1074) were male. The majority of the participants were residents of the central region (706/1074, 65.74%).

Regarding health, only 53.35% (573/1074) thought that their general health was very good or excellent, and 35.20% (378/1074) reported that they never engage in physical activity for at least 15 minutes. A total of 44.51% (478/1074) had a BMI in the normal range, and 48.23% (518/1074) had overweight or moderate or severe obesity. Of respondents, 11.17% (120/1074) were smokers, and only 9.22% (99/1074) reported that a health care provider had recommended a weight management app to them. The most prevalent medical diagnoses that the respondents reported having were depression (69/1074, 6.42%), diabetes (41/1074, 3.82%), and hypertension (29/1074, 2.70%).

A simple logistic regression model was applied to test whether BMI predicted the use of a weight management app. Results revealed that high BMI was significantly associated with the use of a weight management app (𝛸^2^_1_=5.88, *P*<.02). The odds ratio for an increase in BMI was 1.18 (95% CI 1.03-1.35).

**Table 1:** Sociodemographic and health status characteristics of participants stratified by use patterns (n=1074).

Characteristic		User (n=324)	Ex-user (n=195)	Nonuser (n=555)	Total (n=1074)
**Age in years, n (%)**					
	18-31	200 (61.73)	150 (76.92)	435 (78.38)	785 (73.09)
	32-45	108 (33.33)	41 (21.03)	110 (19.82)	259 (24.12)
	≥46	16 (4.94)	4 (2.05)	10 (1.80)	30 (2.79)
**Gender, n (%)**					
	Female	233 (71.91)	144 (73.85)	374 (67.39)	751 (69.93)
	Male	91 (28.09)	51 (26.15)	181 (32.61)	323 (30.07)
**Region of country, n (%)**					
	Central	204 (62.96)	135 (69.23)	367 (66.13)	706 (65.74)
	Southern	14 (4.32)	5 (2.56)	19 (3.42)	38 (3.54)
	Eastern	37 (11.42)	10 (5.13)	43 (7.75)	90 (8.38)
	Northern	33 (10.19)	24 (12.31)	56 (10.09)	113 (10.52)
	Western	33 (10.19)	15 (7.69)	62 (11.17)	110 (10.24)
	Living abroad	3 (0.93)	6 (3.08)	8 (1.44)	17 (1.58)
**Education, n (%)**					
	High school	55 (16.98)	34 (17.44)	106 (19.10)	195 (18.16)
	Bachelor’s degree	207 (63.89)	125 (64.10)	330 (59.46)	662 (61.64)
	Postgraduate	62 (19.14)	36 (18.46)	119 (21.44)	217 (20.20)
**Employment, n (%)**					
	Student	192 (59.26)	126 (64.62)	277 (49.91)	595 (55.40)
	Not employed	21 (6.48)	18 (9.23)	52 (9.37)	91 (8.47)
	Retired	0 (0)	1 (0.51)	11 (1.98)	12 (1.12)
	Employee	111 (34.26)	50 (25.64)	215 (38.74)	376 (35.01)
**Household income per month (SR), n (%)**					
	<5000	175 (54.01)	108 (55.38)	265 (47.75)	548 (51.02)
	5001-10,000	16 (4.94)	20 (10.26)	58 (10.45)	94 (8.75)
	10,001-20,000	59 (18.21)	31 (15.90)	107 (19.28)	197 (18.34)
	>20,001	66 (20.37)	29 (14.87)	104 (18.74)	199 (18.53)
**General health status, n (%)**					
	Excellent	87 (26.85)	41 (21.03)	124 (22.34)	252 (23.46)
	Very good	99 (30.56)	60 (30.77)	162 (29.19)	321 (29.89)
	Good	81 (25.00)	51 (26.15)	151 (27.21)	283 (26.35)
	Average	52 (16.05)	39 (20.00)	106 (19.10)	197 (18.34)
	Poor	5 (1.54)	4 (2.05)	12 (2.16)	21 (1.96)
**Exercise frequency in the past week^a^, n (%)**					
	None	78 (24.07)	75 (38.46)	225 (40.54)	378 (35.20)
	1 day	34 (10.49)	33 (16.92)	91 (16.40)	158 (14.71)
	2 days	60 (18.52)	37 (18.97)	83 (14.96)	180 (16.76)
	3-4 days	88 (27.16)	30 (15.38)	107 (19.28)	225 (20.95)
	5-6 days	64 (19.75)	20 (10.26)	49 (8.83)	133 (12.38)
**Nutrition status of diet, n (%)**					
	Excellent	26 (8.02)	10 (5.13)	23 (4.14)	59 (5.49)
	Very good	73 (22.53)	19 (9.74)	84 (15.14)	176 (16.39)
	Good	103 (31.79)	70 (35.90)	175 (31.53)	348 (32.40)
	Fair	84 (25.93)	66 (33.85)	182 (32.79)	332 (30.91)
	Poor	38 (11.73)	30 (15.38)	91 (16.40)	159 (14.80)
**BMI, n (%)**					
	Underweight	16 (4.94)	14 (7.18)	48 (8.65)	78 (7.26)
	Normal	139 (42.90)	91 (46.67)	248 (44.68)	478 (44.51)
	Overweight	92 (28.40)	54 (27.69)	160 (28.83)	306 (28.49)
	Moderate obesity	50 (15.43)	20 (10.26)	65 (11.71)	135 (12.57)
	Severe obesity	27 (8.34)	16 (8.21)	34 (6.13)	77 (7.17)
**Smoking, n (%)**					
	Yes	32 (9.88)	21 (10.77)	67 (12.07)	120 (11.17)
	No	292 (90.12)	174 (89.23)	488 (87.93)	954 (88.83)
**App recommended by provider, n (%)**					
	Yes	52 (16.05)	26 (13.33)	21 (3.87)	99 (9.22)
	No	265 (81.79)	165 (84.62)	396 (71.35)	826 (76.91)

^a^At least 15 minutes of exercise or physical activity.

### User Perceptions and Use Patterns

Analyses of user perceptions and patterns of use of weight management app are presented in Multimedia Appendix 3. All data were obtained and analyzed from the user group only, where participants reported using a weight management app in the previous 6 months. The frequency of use was 2 or more times per day for 27.8% (90/324) of respondents, once a day for 20.1% (65/324), and a few times each week for 20.1% (65/324). The most common reasons for wanting to download a weight management app were to monitor food intake (319/860, 37.1%) and lose weight (258/860, 30.0%). The most common reasons for downloading a particular weight management app were recommendations from friends and family (153/294, 52.0%), and its rank in the App Store (65/294, 22.1%).

The most reported desirable features were (1) the possibility to be monitored by a specialist (323/976, 33.1%), (2) barcode identification of calorie content (191/976, 19.6%), (3) availability of nutrition information on numerous food items (153/976, 15.7%), (4) weekly or monthly progress report (152/976, 15.6%), and (5) constant reminders to follow a chosen diet or exercise plan (157/976, 16.1%). Most users agreed or strongly agreed that apps that suggested exercise and diet plans helped them lose weight (246/324, 75.9%).

A large proportion of weight management app users agreed or strongly agreed that apps were effective for losing weight (185/324, 57.1%). Regarding accuracy, 48.8% (158/324) of app users believed that apps are accurate, whereas 1.9% (6/324) did not use an app that recorded their data. Of the current weight management app users, only 7.1% (23/324) believed that weight management apps were not secure. A large proportion of app users (180/324, 55.6%) noted that they would never pay anything for a weight management app.

### Reasons for Discontinuing Use

Analyses of the reasons for discontinuing use are presented in Multimedia Appendix 3. Of the participants, 18.16% (195/1074) had downloaded weight management apps that they no longer use. The most reported reasons for discontinuing use were (1) loss of interest (64/195, 32.8%), (2) hidden costs (53/195, 27.2%), (3) monitoring by a specialist was not offered (27/195, 13.8%), (4) difficulty using the app (21/195, 10.8%), and (5) language barrier (18/195, 9.2%).

### Apps that Participants Used for Weight Management

Of the users, 53.2% (179/324) listed the name of the apps they used (Multimedia Appendix 4). However, because the question on app name was open-ended, some participants wrote ambiguous names or cited more than one app. The total number of weight management apps mentioned by users (more than once) was 267. The most reported apps were MyFitnessPal (145/267, 54.3%), health apps that come with a smartphone (16/267, 6.0%), StepsApp Pedometer (13/267, 4.9%), Soarrate (10/267, 3.8%), and Fitbit (10/267, 3.8%).

### App Search

**Figure 1:**
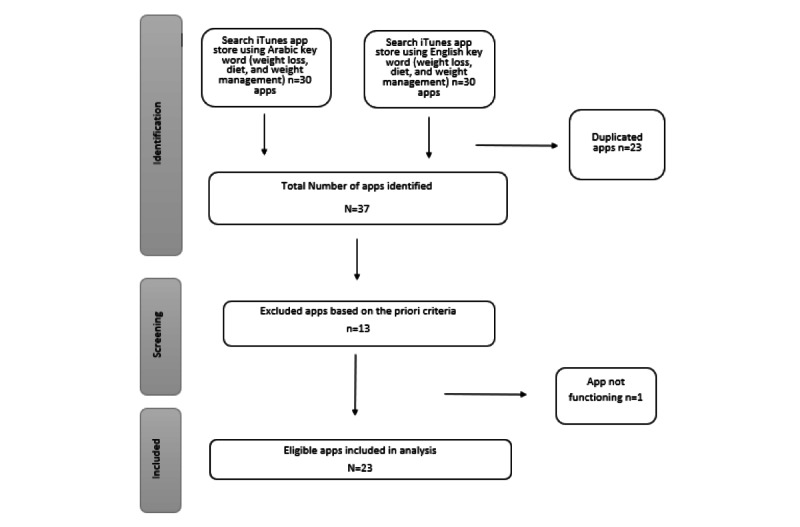
Flow diagram of app search.

A total of 60 apps were identified from the search in the Saudi App Store. Of these apps, we excluded 23 that were duplicates, 12 that were not free, 1 app that did not function, and 1 app that appeared but was described in the app store as a book. Figure 1 provides a description of the search process.

A total of 23 apps met the inclusion criteria. The app ratings in the App Store varied from 2.1 to 5 (out of 5). Of these, 65% (15/23) received 4 or more stars, and the number of users that rated each app were 1000 or more (5/15, 33%), 100 to 1000 (5/15, 33%), or less than 100 (5/15, 33%). Of the apps, 48% (11/23) were updated by the app developers 1 day to 2 months after data collection. Of the apps, 52% (12/23) were only available in Arabic and, among these, 25% (3/12) were apps for purchase. Approximately 30% (7/23) of the apps were available in English, and 86% (6/7) of these were apps for purchase. Only 17% (4/23) of the apps were available in both English and Arabic (Multimedia Appendix 5).

### App Quality Scores

#### Mobile App Rating Scale

Table 2 presents the final scores, means, and standard deviations for the following: (1) 4 subscales (engagement mean, functionality mean, aesthetics mean, and information mean), (2) overall quality (mean of 4 subscales), and (3) subjective quality of the 23 apps. The average MARS quality of the 23 apps was acceptable, and scores varied from 1.7 to 4.4 (out of 5.0). The reliability of the objective scales calculated as Cronbach alpha = .95. The average MARS quality score for the apps was 3.3 (SD 0.8) out of 5.0. The mean of the subjective scores was 2.5 (SD 1.1). Of the 4 subscales, functionality had the highest median score of 3.6 (SD 0.8), and information had the lowest median score of 2.9 (SD 0.9). Of the apps, 30% (7/23) received a quality mean score of 4.0 or higher; only one of these apps was originally an Arabic app. Of the apps, 30% (7/23) did not meet the acceptability score of 3.0 or higher, and all of these were in the Arabic language only (Table 2).

**Table 2:** The Mobile App Rating Scale mean scores for weight management apps^a^.

App	Engagement	Functionality	Aesthetics	Information	MARS^b^	Subjective
Lose Weight for Men	4.4	5.0	4.3	4.0	4.4	4.0
Rashaqa adad alsoarat	4.6	4.8	4.7	3.5	4.4	3.8
MyFitnessPal	4.4	4.0	4.3	4.4	4.3	4.3
Fitbit	4.4	4.3	4.7	3.7	4.3	3.5
StepsApp Pedometer	3.8	4.8	4.3	3.8	4.2	2.8
Lose It! – Calorie Counter	4.4	4.0	4.0	3.4	4.0	3.3
Calorie Counter by FatSecret	4.2	4.3	3.7	3.6	4.0	4.0
Pacer Pedometer	4.8	3.5	3.7	3.5	3.9	3.8
Lifesum – Diet & Food Diary	4.2	3.8	4.0	3.5	3.9	3.0
7 Minute Workout: Fitness App	3.8	4.0	4.3	3.6	3.9	3.0
FUDC – Follow-Up Diet and Calories	4.2	4.0	3.3	2.8	3.6	2.5
mDiet	3.0	4.0	3.0	3.2	3.3	2.5
Adaad alsoaraat	4.2	3.0	3.0	3.1	3.3	2.3
Soarrate	3.4	3.3	3.0	3.6	3.3	2.5
Weight Tracker	3.2	3.8	2.6	2.5	3.0	2.5
My Diet Coach – Weight Loss	3.4	3.0	2.6	3.1	3.0	1.8
Tmarin manzliah	3.0	3.8	2.3	2.3	2.9	1.3
Alwazan almethali	2.6	3.0	3.0	2.4	2.8	1.3
Hesab alwazan almethali	2.6	3.0	3.0	1.6	2.6	1.3
Monabeh alsoaraat	2.6	2.5	2.3	1.8	2.3	1.3
Diet	1.8	2.8	2.0	2.0	2.2	1.3
Rajeem 7kilo fi esboaa	1.4	2.5	1.6	1.2	1.7	1.0
Rajem sareea	1.4	2.5	1.6	1.2	1.7	1.0
Subscales mean (SD)	3.5 (1.0)	3.6 (0.8)	3.3 (1.0)	2.9 (0.9)	3.3 (0.8)	2.5 (1.1)

^a^All items were rated on a 5-point scale from 1=inadequate to 5=excellent.

^b^MARS: Mobile App Rating Scale.

The MARS scores for the apps available only in the Arabic language ranged between 1.7 and 4.4 and for apps available in only English, the scores ranged between 3.0 and 4. Apps available in English and Arabic had scores that ranged between 3.0 and 4.4 (Multimedia Appendix 6).

**Figure 2:**
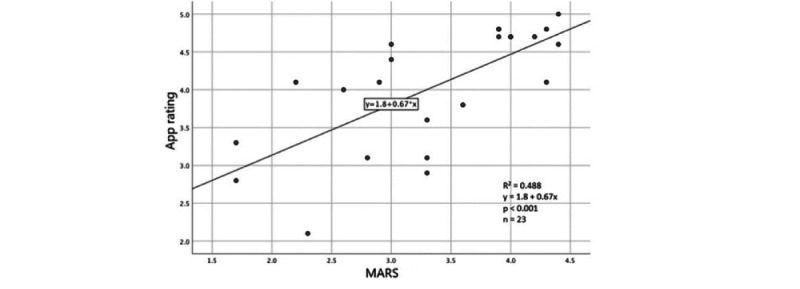
Regression analyses of the association between the star rating in the App Store and the Mobile App Rating Scale score.

A simple linear regression was applied to predict the relation between the star rating in the App Store and the MARS score and showed a significant association (F_1,21_=20.018, *P*<.001) with an *R*² of .488 (Figure 2).

For engagement and aesthetics, Lose Weight for Men, Rashaqa adad alsoarat, MyFitnessPal, Fitbit, Lose It! – Calorie Counter, and Lifesum – Diet & Food Diary were the highest rated apps. Despite the high functionality of the Arabic weight management apps, Multimedia Appendix 6 shows that the lowest engagement mean was for Arabic apps.

Of the 23 apps, 14 were mentioned more than once by app users in the survey; 11 apps were found in the survey responses but did not appear in our app search. Multimedia Appendix 7 shows the MARS mean scores of the apps and the number of users who reported using the apps.

### Assessment of Evidence-Based Strategies

Table 3 provides the frequency of evidence-based strategies within the included apps. The most common strategies were as follows (no free apps or versions offered personalized feedback to the user):

- Self-monitoring (17/23, 74%) allowed the user to track weight and/or physical activity over time. A few apps included more comprehensive tracking options such as for nutrition, sleep, and cardiometabolic indicators
- Weight and/or health assessment features (17/23, 74%) enable the app to assess the BMI and/or calorie requirement
- Goal setting (13/23, 57%) mainly consisted of goals for weight loss, calorie balance, water intake, or physical activity
- Healthy eating support (12/23, 52%) was mostly in the form of healthy eating guidelines, meal plans, and nutritional information on specific foods
- Physical activity support (9/23, 39%) including physical activity tips and plans
- Motivational strategies (7/23, 30%) included prompts, gamification, or use of rewards such as points for meeting weight goals or physical activity levels
- Social support components (8/23, 35%) included the ability to communicate online with other users

Of the apps that were available in Arabic, 50% (6/12) had one or two EBS features, 17% (2/12) had 3 features, 25% (3/12) had 5 features, and 8% (1/12) had 6 features. Of these, 75% (9/12) of the apps provided weight/health assessment and 66% (8/12) provided healthy eating support. Feedback was not observed in any of the apps, and motivation strategy was observed in 1 app.

Self-monitoring, goal setting, and weight/health assessment were observed in the majority of apps (3/4, 75%) available in English and Arabic. However, the percentages for healthy eating, social support, and feedback were 25% (1/4), 25% (1/4), and 0%, respectively.

Of the apps not available in Arabic, 43% (3/7) had 3 features, 29% (2/7) had 5 features, and 29% (2/7) had 7 features. Of these, 100% (7/7) of the apps provided self-monitoring and 71% (5/7) provided goal setting, weight/health assessment, and motivation strategy.

**Table 3:** Assessment of evidence-based strategies for weight management apps^a^.

App name	Self-monitoring	Goal setting	Healthy eating support	Physical activity support	Social support	Weight /health assessment	Motivational strategies	Personalized feedback	Total EBS^b^ within app (n=8) n (%)
MyFitnessPal	1	1	1	1	1	1	1	0	7 (88)
Fitbit	1	1	1	1	1	1	1	0	7 (88)
Rashaqa adad alsoarat	1	1	0	1	1	1	1	0	6 (75)
FUDC - Follow-Up Diet and Calories	1	1	1	0	1	1	0	0	5 (63)
Pacer Pedometer	1	1	0	1	1	0	1	0	5 (63)
Calorie Counter by FatSecret	1	1	1	0	1	1	0	0	5 (63)
Adaad alsoaraat	1	1	1	1	0	1	0	0	5 (63)
Soarrate	1	1	1	0	1	1	0	0	5 (63)
Weight Tracker	1	1	1	0	0	1	0	0	4 (50)
Lose It! - Calorie Counter	1	1	0	0	1	1	0	0	4 (50)
My Diet Coach - Weight Loss	1	1	0	0	0	0	1	0	3 (38)
mDiet	0	1	1	0	0	1	0	0	3 (38)
Lifesum - Diet & Food Diary	1	0	0	0	0	1	1	0	3 (38)
Tmarin manzliah	1	0	0	1	0	1	0	0	3 (38)
7 Minute Workout: Fitness App	1	0	0	1	0	1	0	0	3 (38)
Lose Weight for Men	1	0	0	1	0	1	0	0	3 (38)
StepsApp Pedometer	0	1	0	1	0	0	1	0	3 (38)
Alwazan almethali	1	0	0	0	0	1	0	0	2 (25)
Diet	0	0	1	0	0	1	0	0	2 (25)
Hesab alwazan almethali	1	0	0	0	0	1	0	0	2 (25)
Monabeh alsoaraat	0	0	1	0	0	0	0	0	1 (13)
Rajeem 7kilo fi esboaa	0	0	1	0	0	0	0	0	1 (13)
Rajem sareea	0	0	1	0	0	0	0	0	1 (13)
Total apps	17	13	12	9	8	17	7	0	N/A^c^

^a^1=presence, 0=absence.

^b^EBS: evidence-based strategies.

^c^N/A: Not applicable.

**Figure 3:**
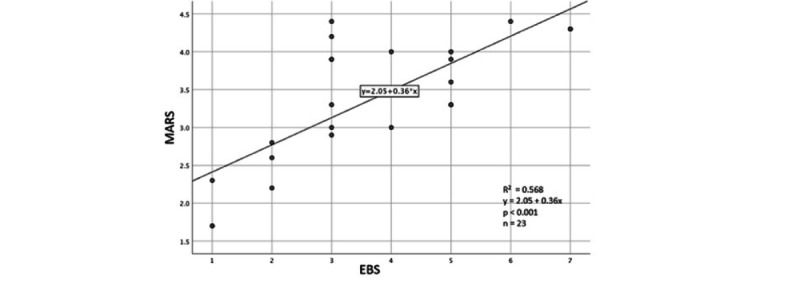
Regression analyses of the association between the Mobile App Rating Scale score and the evidence-based strategy criteria.

A simple linear regression was calculated to predict the MARS scale based on EBS. A significant regression equation was found (F_1,21_=27.66, *P*<.001), with an *R*² of .568 (Figure 3). Multimedia Appendix 8 shows the EBS scores of the apps and the number of users who reported using the apps.

### Six Sigma and the Process Capability Index

Table 4 shows the Cpk calculations for each of the 23 apps found in the App Store and their sigma levels. Note that we used the limits mean in Cpk calculations. This is because a calculation based on data mean mostly provides a negative Cpk, which leads to the conclusion that the score levels of MARS are irrelevant to the actual app quality. Therefore, we adjusted the analysis assumption to provide results that are more relevant.

The quality thresholds of the two popular measurements tools, MARS and EBS, used in this analysis are presented in Table 5. Six sigma was used to evaluate the thresholds of these tools based on data from Table 2, Table 3, and Multimedia Appendix 4.

The calculation for the EBS Cpk was as follows: µ=3.608695652, σ=1.777105165, and Cpk=–0.073397335. The calculation for the MARS Cpk was as follows: µ=3.347826087, σ=0.845775365, and Cpk=0.137083715.

The sigma results indicate that EBS has no level, as its Cpk is negative. This means that most of the apps failed to pass the mean indicating that newly developed apps will probably fail as well. The sigma level of MARS at level 1 means that their evaluation criteria are more realistic and closer to the natural structure of weight management apps.

**Table 4:** Six sigma behavioral evaluation for the quality model: engagement, functionality, aesthetics, and information.

App name	Engagement	Functionality	Aesthetics	Information	µ^a^	σ^b^	Cpk^c^	Sigma level
Pacer Pedometer	4.8	3.5	3.7	3.5	3.88	0.62	0.53	2
Rashaqa adad alsoarat	4.6	4.8	4.7	3.5	4.40	0.61	0.55	2
Lose It! - Calorie Counter	4.4	4.0	4.0	3.4	3.95	0.41	0.81	3
Fitbit	4.4	4.3	4.7	3.7	4.28	0.42	0.79	3
Lose Weight for Men	4.4	5.0	4.3	4.0	4.43	0.42	0.79	3
MyFitnessPal	4.4	4.0	4.3	4.4	4.28	0.19	1.76	6
Adaad alsoaraat	4.2	3.0	3.0	3.1	3.33	0.59	0.57	2
FUDC - Follow-Up Diet and Calories	4.2	4.0	3.3	2.8	3.58	0.64	0.52	2
Calorie Counter by FatSecret	4.2	4.3	3.7	3.6	3.95	0.35	0.95	3
Lifesum - Diet & Food Diary	4.2	3.8	4.0	3.5	3.88	0.30	1.12	4
StepsApp Pedometer	3.8	4.8	4.3	3.8	4.18	0.48	0.70	3
7 Minute Workout: Fitness App	3.8	4.0	4.3	3.6	3.93	0.30	1.12	4
Soarrate	3.4	3.3	3.0	3.6	3.33	0.25	1.33	4
My Diet Coach - Weight Loss	3.4	3.0	2.6	3.1	3.03	0.33	1.01	4
Weight Tracker	3.2	3.8	2.6	2.5	3.03	0.60	0.55	2
Tmarin manzliah	3.0	3.8	2.3	2.3	2.85	0.71	0.47	2
mDiet	3.0	4.0	3.0	3.2	3.30	0.48	0.70	3
Hesab alwazan almethali	2.6	3.0	3.0	1.6	2.55	0.66	0.50	2
Monabeh alsoaraat	2.6	2.5	2.3	1.8	2.30	0.36	0.94	3
Alwazan almethali	2.6	3.0	3.0	2.4	2.75	0.30	1.11	4
Diet	1.8	2.8	2.0	2.0	2.15	0.44	0.75	3
Rajeem 7kilo fi esboaa	1.4	2.5	1.6	1.2	1.68	0.57	0.58	2
Rajem sareea	1.4	2.5	1.6	1.2	1.68	0.57	0.58	2

^a^µ: data mean.

^b^σ: standard deviation of the sample data.

^c^Cpk: process capability index.

**Table 5:** Comparison of app quality according to mobile users (survey outcomes), evidence-based strategy, and the Mobile App Rating Scale.

App name	Limits 4.0-8.0 EBS^a^	Limits 3.0-5.0 MARS^b^	Survey	Within limit of N≥50 EBS	Within limit of acceptable=3 MARS
MyFitnessPal	7.0	4.3	54.3	TRUE	TRUE
Fitbit	7.0	4.3	3.8	TRUE	TRUE
Rashaqa adad alsoarat	6.0	4.4	1.5	TRUE	TRUE
FUDC - Follow-Up Diet and Calories	5.0	3.6	0	TRUE	TRUE
Pacer Pedometer	5.0	3.9	0.7	TRUE	TRUE
Calorie Counter by FatSecret	5.0	4.0	1.9	TRUE	TRUE
Adaad alsoaraat	5.0	3.3	3.0	TRUE	TRUE
Soarrate	5.0	3.3	3.8	TRUE	TRUE
Weight Tracker	4.0	3.0	0.7	TRUE	TRUE
Lose It! - Calorie Counter	4.0	4.0	3.4	TRUE	TRUE
My Diet Coach - Weight Loss	3.0	3.0	0.7	FALSE	TRUE
mDiet	3.0	3.3	2.2	FALSE	TRUE
Lifesum - Diet & Food Diary	3.0	3.9	3.0	FALSE	TRUE
Tmarin manzliah	3.0	2.9	0	FALSE	FALSE
7 Minute Workout: Fitness App	3.0	3.9	0	FALSE	TRUE
Lose Weight for Men	3.0	4.4	0	FALSE	TRUE
StepsApp Pedometer	3.0	4.2	4.9	FALSE	TRUE
Alwazan almethali	2.0	2.8	0.7	FALSE	FALSE
Diet	2.0	2.2	0	FALSE	FALSE
Hesab alwazan almethali	2.0	2.6	0	FALSE	FALSE
Monabeh alsoaraat	1.0	2.3	0	FALSE	FALSE
Rajeem 7kilo fi esboaa	1.0	1.7	0	FALSE	FALSE
Rajem sareea	1.0	1.7	0	FALSE	FALSE

^a^EBS: evidence-based strategy.

^b^MARS: Mobile App Rating Scale.

## Discussion

### Principal Findings

In this survey, we identified user perceptions of weight management apps and reasons for using such apps. We also assessed the quality of weight management apps available at the Saudi App Store using the MARS quality score, an EBS assessment, and six sigma evaluations. The user surveys and evaluation of features indicated that personalized feedback is the most common feature lacking among commercial apps, and it is the feature that users most desired. According to the majority of ex-users, the reasons for stopping their use of weight management apps included a loss of interest and hidden costs.

Regarding app appraisal, the EBS and MARS quality scores showed that the quality of weight management app features was variable. The behavior of a weight management app in all 4 MARS quality attributes predicts its behavior toward other quality attributes, and as a result determines its overall quality based on stronger judgment than the mean only. For instance, Rashaqa adad alsoarat had the second best score in MARS but only achieved level 2 as its behavior to the different quality attributes was variable. MyFitnessPal was not the best in MARS; however, it achieved level 6 because of consistent behavior in all 4 quality attributes, resulting in 54% of weight management app users using MyFitnessPal.

Six sigma evaluations indicated that several apps have high scores for engagement and functionality, but these are not matched with MARS. The six sigma results lead us to the following question: If we apply a MARS or EBS evaluation to an app, does this indicate that the app will be used by mobile users?

Measurement tools like MARS and EBS evaluate software based on the mean score only. More specifically, if the app passes the mean, it is assumed to be of good quality, and good quality apps should find their way to the market, but this is not the case. In fact, passing the mean does not ensure the quality is consistently distributed through all of the app’s quality properties, and in turn that the app has good quality. Using MARS only allows an app to score high if it meets one out of the 4 quality attributes of MARS. However, the sigma results of EBS reflect that most of the apps failed to pass the mean, indicating that newly developed apps will likely fail. This stresses the importance of reevaluating the passing criterion, which is ≥50%. We can infer from Multimedia Appendix 7 and 8 that neither the MARS nor the EBS tool gives developers an indication of the acceptance of their app by mobile users. Thus, based on Cpk results, six sigma is a better tool to identify the quality of a weight management app and if it actually meets MARS quality attributes.

### Comparison With Prior Work

We targeted the general population to approach different types of users. This approach was previously adopted in studies in the United States [9], Saudi Arabia [11], Germany [23], and China [24]. In our study, 30% of the survey participants were app users, a percentage that is lower than in other studies [9,11,24], possibly because we excluded users who had not used the app in the previous 6 months, unlike in other studies. We found that the highest use of weight management apps was among women; similar studies have also found that women are more involved in weight control and healthy eating than men [25,26].

The majority of users believe that weight management apps are effective. The effectiveness of weight management apps was established in a systematic review and meta-analysis of 8 randomized controlled trials comparing the use of weight management apps for weight loss to traditional care or intensive consulting [10]. The systematic review and meta-analysis results indicated a significant effect of weight management apps through a 1 kg reduction in body weight.

To the best of our knowledge, evaluating weight management apps in Arabic using EBS has not been done previously. However, in 2016, Arabic apps were evaluated based on 13 evidence-informed practices [27]; the difference between these two tools is that EBS represents broader criteria than evidence-based practices. The advantage of using EBS is in describing the overarching evidence-based quality of the current market for weight management apps. Evaluating Arabic apps using different strategies from before extends the current literature. In our sample, the most desirable features reported by the app users are the possibility to be monitored by a specialist and barcode identification of calorie content. These features reflect two strategies of evidence-based features, which are personalized feedback and healthy eating support [17].

The average number of evidence-based features present in an app was between 3 and 4, which was more than in a previous study [17] and could be explained by the fact that app content is improving over time. In our findings, the most popular feature is self-monitoring, which is consistent with the findings of previous studies [17]. In contrast, the majority of the apps in the Saudi App Store lack the personalized feedback feature.

Weight management apps in Arabic have limited strategies. However, a comparison with a study conducted on Arabic weight management apps found that our results show improvements in the Arabic apps [27]. Overall, weight management features of apps found in the Saudi App Store have weak adherence to EBS, which may be the result of the lack of health care expert involvement during app development.

In our study, the average MARS quality scores for the 23 weight management apps available in the App Store varied significantly, with 7 apps not meeting the minimum acceptability score of 3.0. None of the apps received the maximum score of 5.0. These findings are similar to that of a previous study that examined 23 weight management apps available in the App Store and Google Play [28]. However, the maximum quality score was higher than our findings, which may be because of the inclusion of paid apps [28].

The weakest MARS subscale was for the quality of information. In contrast, the functionality subscale had the highest median score, a result that is in line with previous studies that used MARS to assess the quality of mindful-eating mobile apps [14] and weight management apps [28]. Another study that evaluated apps for managing tinnitus [28] also found that functionality had the highest MARS subscale.

MARS indicates that some apps, like the StepsApp Pedometer, have high scores for engagement and functionality that are not reflected in the EBS results. Several apps have powerful features and efficiency but require careful future evaluation for long-term use.

In our survey, the MyFitnessPal app was the most cited by the participants. This app was mentioned more than 140 times, and the second most cited app was mentioned only 16 times. Furthermore, the assessment of the quality and features of weight management apps showed that MyFitnessPal has good quality traits and 88% of the EBS. Other weight management apps had equal quality and evidence-based features but were less popular among the participants. This lack of popularity could be the result of other factors that impact app popularity. For example, we asked app users about their reasons for downloading a specific app from the App Store. The most reported reason was recommendations from friends or relatives. Therefore, app users recommending a particular app to their friends and relatives could cause a snowball effect and lead to an increase in the use of a particular app.

### Strengths and Limitations

Our study, which included a large number of participants across Saudi Arabia, shows the overall quality of weight management apps, reports on the areas of the apps that are weak or strong, and notes the app strategies that are absent or present. Such information can assist app developers in enhancing their current apps or developing new, better apps.

Although this study represents the first appraisal of weight management apps downloaded from the App Store in Saudi Arabia, we only included free apps or free versions. Because Android apps were not reviewed, and the app search was limited to the Saudi App Store, the findings cannot be generalized to all smartphone apps. As a result, the possibility exists that we missed additional apps or features. Also, MARS includes items that may be a source of subjective bias; however, having two independent reviewers applying MARS, with a third reviewer resolving discrepancy, helped reduce bias, and the same method was used when applying EBS. Another limitation of our study is that the survey data are self-reported, which could be a source of error. Furthermore, classifying participants based on BMI categories does not reflect body composition, therefore, we were unable to report results on fat and fat-free mass. In addition, our study was limited by the recruitment strategy, so we may have missed individuals with limited internet access or those not using social media.

### Conclusions

Despite the large quantity and easy accessibility of weight management apps, the quality and features of the majority of apps from the App Store included in the study remains low. Improvements made to Arabic apps have been limited, and the information content needs to be enhanced. In general, we found that the weakest areas of apps from the App Store are information quality and graphic design. App users wanted a feature that allows them to communicate with a specialist, so this feature should be considered by app developers in the future. Additionally, we can infer that MARS and EBS do not give developers an indication of the acceptance of their apps by mobile users. This stresses the importance of reevaluating the passing criterion and approaching users when developing an app. Our findings lead to the recommendations that significant attention should be paid to supporting the maintainability of weight management apps in the future.

## Appendix

Multimedia Appendix 1 Checklist for reporting results of internet e-surveys (CHERRIES).

Multimedia Appendix 2 Questionnaire: weight-management apps in Saudi Arabia: feature assessment, and quality evaluation.

Multimedia Appendix 3 Users perception, pattern of use, and reasons for discounting use.

Multimedia Appendix 4 Apps that participants used for weight management (n=267).

Multimedia Appendix 5 Weight management apps and their ratings in the App Store.

Multimedia Appendix 6 The Mobile App Rating Scale mean scores for weight management apps stratified by app language.

Multimedia Appendix 7 The Mobile App Rating Scale mean scores and the number of users who reported using the apps.

Multimedia Appendix 8 Evidence-based strategy assessment scores and the number of users who reported using the apps.

## Abbreviations

CHERRIES: Checklist for Reporting Results of Internet e-Surveys
Cpk: process capability index
EBS: evidence-based strategy
LSL: lower limit of customer expectations
MARS: Mobile App Rating Scale
mHealth: mobile health
USL: upper limit of customer expectations
µ: data mean
σ: standard deviation of the sample data
