# Supplementary material for: Weight Management Apps in Saudi Arabia: Evaluation of Features and Quality
Source: JMIR Mhealth Uhealth. 2020 Oct 26;8(10):e19844. doi: 10.2196/19844 (PMC7652688; doi:10.2196/19844)
Supplement: Multimedia Appendix 1 [file mhealth_v8i10e19844_app1.docx]

**Multimedia Appendix 1. Checklist for reporting results of internet e-surveys (CHERRIES)**

| Item category | Checklist item | Page no. | Description |
| --- | --- | --- | --- |
| Design | Study design | 4 | A cross-sectional web-based survey was conducted with Saudi smartphone users. Over four months, smartphone users were invited to participate in an anonymous, web-based survey hosted on a Microsoft platform. |
| Ethics | Ethics approval | 4 | The King Saud University Institutional Review Board approved the study (reference E-19-4001) in May 2019. |
|  | Informed consent | 4 | Informed consent was a requirement for participation |
|  | Data protection | 4 | No personal identifying information was collected |
| Development and  Pre-testing |  | 4 | The questions were obtained from questionnaires used in the United States [9].  The questionnaire was tested on a small sample of app users and nonusers (n=10). Depending on the feedback and results from the test, the questions and question order were revised to reduce response bias and enhance response time.  The final questionnaire consisted of 31 questions. The survey was comprised of a mix of open and closed questions and took between five and nine minutes to complete. |
| Recruitment process | Open vs closed  survey | 4 | The survey was open to anyone who would like to take part and both app users and nonusers were invited. |
|  | Contact mode | 4 | The survey link was advertised through social media and university portals. |
|  | Advertising the  survey | 4 | Twitter ads were used to promote the survey link in eight Saudi cities, including Baha, Eastern, Tabuk, Asir, Makkah, Almadeinah, Hail, and Jazan. The ad ran during the second week of September 2019 for seven days. |
| Survey  administration | Web/email | 4 | The survey was an online survey which was hosted on a Microsoft platform. |
|  | Context | 4 | The survey was an online survey which was hosted on a Microsoft platform. The survey link was advertised through social media and university portals. |
|  | Mandatory/voluntary | 4 | The survey was open to anyone who  would like to take part and both app  users and non-users were invited. |
|  | Incentives |  | Not applicable |
|  | Time/date | 4 | Responses were collected between June 2019 and September 2019. |
|  | Item randomisation |  | No randomisation of items was used. |
|  | Adaptive Questioning |  | Not applicable |
|  | Number of items | 4 | The final questionnaire consisted of 31 questions. |
|  | Number of screens | 4 | The final web-based questionnaire was presented on three screens. |
|  | Completeness  check | 7 | A total of 1,209 people responded to the survey. The data were excluded from the analysis if the respondents were non-Saudi, younger than 18 years, provided inconsistent or illogical answers (i.e., participant reported a weight of –10 kg), or were app users and failed to answer more than 50% of the questions on app usage. |
|  | Review step | 4 | The back icon on each screen allowed subjects to edit previous answers. But  respondents were unable to change  their responses once submitted. |
| Response Rates | Unique site visitor | 4 | Only one submission from each IP  address was permitted by the survey  software. |
|  | View rate |  | Not collected |
|  | Participation rate | 7 | A total of 1,209 people responded to the survey. Of the participants who read the welcome page and proceeded to consent, 1,193 agreed to participate in the survey, which translated into a 98.7% (1,193/1,209) participation rate. |
|  | Completion rate | 7 | The data were excluded from the analysis if the respondents were non-Saudi, younger than 18 years, provided inconsistent or illogical answers (i.e., participant reported a weight of –10 kg), or were app users and failed to answer more than 50% of the questions on app usage. There were 1,074 remaining responses for further analysis. |
|  | Cookies used |  | No |
|  | IP Check | 4 | Only one submission from each IP  address was permitted by the survey  software. |
|  | Log file analysis |  | No |
|  | Registration |  | Not applicable |
| Analysis | Handling of  incomplete  questionnaires | 7 | The data were excluded from the analysis if the respondents were app users and failed to answer more than 50% of the questions on app usage. There were 1,074 remaining responses for further analysis. |
|  | Questionnaires  with atypical  timestamp |  | Not applicable |
|  | Statistical  correction |  | Not applicable |
