# Supplementary material for: Weight Management Apps in Saudi Arabia: Evaluation of Features and Quality
Source: JMIR Mhealth Uhealth. 2020 Oct 26;8(10):e19844. doi: 10.2196/19844 (PMC7652688; doi:10.2196/19844)
Supplement: Multimedia Appendix 2 [file mhealth_v8i10e19844_app2.docx]

**Multimedia Appendix 2. Questionnaire: weight-management apps in Saudi Arabia: feature assessment, and quality evaluation**

We are inviting you to take a survey for research. The King Saud University Institutional Review Board approved the research (reference E-19-4001) in May 2019.

The purpose of the survey is to explore the use of weight management apps, users’ perceptions, explore reasons for starting, and stop app use.

This survey will take about nine minutes of your time; the participation is entirely voluntary. There are no negative consequences if you do not want to take it. We won’t collect any personal information i.e., your name, email, or IP address. All the survey data will be secured, and it will be used for scientific use only.

If you agree to participate, please choose agree.

- Agree
- Disagree

1. **Nationality**

- Saudi
- Non-Saudi

1. **Gender**

- Female
- Male

1. **Age**

- **- - - - - - -**

1. **Region of Country**

- Central
- Southern
- Eastern
- Northern
- Western
- Living abroad

1. **Education**

- High school degree
- Bachelor’s degree
- Graduate degree (Masters, PhD, MD, etc.)

1. **Employment**

- Student
- employee
- Not employed
- Retired

1. **Household income**

- Less than 5,000 SR/month
- 5,001 to 10,000 SR/month
- 10,001 to 20,000 SR/month
- >20,0001 SR/month

1. **Weight**

- - - - - - - -

1. **Hight**

- - - - - - - -

1. **In general, would you say your health is:**

- Poor
- Average
- Good
- Very good
- Excellent

1. **In the past week, on how many days do you exercise or participate in physical activities for at least 15 minutes?**

- Never
- 1 day
- 2 days
- 3-4 days
- 5-7 days

1. **In general, how healthy is your overall diet?**

- Poor
- Fair
- Good
- Very good
- Excellent

1. **Do you smoke cigarettes?**

- Yes
- No

1. **Have you ever been diagnosed with any of the following conditions by a health professional?**

- None
- Hypercholesterolemia
- Hypertension
- Depression
- Diabetes
- Other chronic disease

1. **Has a healthcare provider ever recommended you use a weight-management app?**

- Yes
- No

1. **Are there weight management apps you downloaded and no longer used?**

- Yes
- No

1. **If your answered in Q16 were (yes) please choose all the reasons (you can choose more than one answer):**

- loss of interest
- hidden costs
- monitoring by a specialist was not offered
- difficulty of using the app
- language barrier

1. **Have you downloaded a mobile app to track anything related to your weight in the past 6 months?**

- Yes
- No

** If your answered in Q18 were (yes) please answer the following questions:

1. **Please list all the weight-management mobile app/s that you have been used in the past 6 months:**

- - - - - - - - -
- - - - - - - - -

1. **Choose all the reasons for downloading a particular weight-management mobile app** **(you can choose more than one answer):**

- Best ranked in the app store
- Recommendations from friends or family
- Social Media influencers
- Web searches (e.g. Google)
- Recommended by other apps
- TV

1. **What would be the maximum amount you would pay for a weight-management mobile app?**

- I wouldn't pay anything
- 15 SR or less
- 16 SR to 22 SR
- More than 23 SR

1. **Choose all the Reason for downloading a weight-management mobile app (you can choose more than one answer):**

- Weight loss
- Monitor food intake
- Track how much activity or exercise I get
- Show or teach me exercises
- I want to kill time when bored

1. **Choose all the features that are available within the weight management mobile app that you have been used (you can choose more than one answer):**

- Monitored by a specialist
- Can identify calories using barcode
- Nutrition Information of many food items
- Provides a weekly or monthly progress report
- Reminders to follow the diet or exercise

1. **Choose all the desired features of weight management mobile apps (you can choose more than one answer).**

- Monitored by a specialist
- Can identify calories using barcode
- Provides Nutrition Information of many food items
- Provide weekly or monthly progress report
- Reminders to follow the diet or exercise

1. **How many weight-managements mobile apps do you use?**

- 1-5
- 6-10
- 11 or more

1. **On average, how often do you open or log on to use the weight-management mobile app?**

- Two or more time a day
- One time each day
- A few times each week
- A few times a month
- Less than once a month

1. **Apps that provide a diet plan helped in managing weight:**

- Strongly disagree
- Disagree
- Strongly agree
- Agree
- Unsure

1. **Apps that provide an exercise plan helped in managing weight:**

- Strongly disagree
- Disagree
- Strongly agree
- Agree
- Unsure

1. **Weight-management mobile apps record user’s data accurately:**

- Strongly disagree
- Disagree
- strongly support
- Support
- Neutral
- I don’t use apps that record my health information

1. **Weight-management mobile apps are effective for long-term use:**

- Strongly disagree
- Disagree
- strongly support
- Support
- Neutral

1. **Weight-management mobile apps keep user’s data safe and secure:**

- Strongly disagree
- Disagree
- strongly support
- Support
- Neutral
