# Supplementary material for: Weight Management Apps in Saudi Arabia: Evaluation of Features and Quality
Source: JMIR Mhealth Uhealth. 2020 Oct 26;8(10):e19844. doi: 10.2196/19844 (PMC7652688; doi:10.2196/19844)
Supplement: Multimedia Appendix 3 [file mhealth_v8i10e19844_app3.docx]

**Multimedia Appendix 3. Users perception, pattern of use, and reasons for discounting use**

| Pattern of use | Total  n (%) | |
| --- | --- | --- |
| Number of weight-management apps used, n (%). (N=324) | | |
| 1-5 apps | 304 (93.8%) | |
| 6-10 apps | 9 (2.8%) | |
| ≥11 apps | 7 (2.2%) | |
| Frequency of weight-management app use, n (%). (N=324) | | |
| Two or more times a day | 90 (27.8%) | |
| One time each day | 65 (20.1%) | |
| A few times each week | 65 (20.1%) | |
| A few times a month | 50 (15.4%) | |
| Less than once a month | 47 (14.5%) | |
| Reasons for downloading a weight-management app, n (%) | |  |
| Weight loss | 258 (30.0%) |  |
| Monitor food intake | 319 (37.1%) |  |
| Track how much activity or exercise I get | 120 (14.0%) |  |
| Show or teach me exercises | 129 (15.0%) |  |
| I want to kill time when bored | 34 (4.0%) |  |
| Reasons for downloading a particular weight-management app, n (%) | |  |
| Best ranked in the app store | 65 (22%) |  |
| Recommendations from friends or family | 153 (52%) |  |
| Social media influencers | 35(11.9%) |  |
| Recommended by other apps | 38 (12.9%) |  |
| TV | 3 (1%) |  |
| Desired features of weight-management apps, n (%) | |  |
| Monitored by a specialist | 323 (33.1%) |  |
| Can identify calories using barcode | 191 (19.6%) |  |
| Provides nutritional information of many foods | 153 (15.7%) |  |
| Provide weekly or monthly report | 152 (15.6%) |  |
| Reminders to follow a diet or exercise | 157 (16.1%) |  |
| Features that are available within your app, n (%) | | |
| Monitored by a specialist | 31 (4.7%) | |
| Can identify calories using barcode | 180 (27.3%) | |
| Nutrition information of many food items | 175 (26.5%) | |
| Provides a weekly or monthly report | 144 (21.8%) | |
| Reminders to follow a diet or exercise | 130 (19.7%) | |
| Apps that provide a diet plan helped in managing weight, n (%). (N=324) | |  |
| Strongly disagree | 4 (1.2%) |  |
| Disagree | 8 (2.5%) |  |
| Strongly agree | 154 (47.5%) |  |
| Agree | 92 (28.4%) |  |
| Unsure | 62 (19.1%) |  |
| Apps that provide an exercise plan helped in managing weight, n (%). (N=324) | |  |
| Strongly disagree | 4 (1.2%) |  |
| Disagree | 8 (2.5%) |  |
| Strongly Agree | 154 (47.5%) |  |
| Agree | 92 (28.4%) |  |
| Unsure | 62 (19.1%) |  |
| Weight-management apps are effective for long-term use, n (%). (N=324) | |  |
| Strongly disagree | 3 (0.9%) |  |
| Disagree | 33 (10.2%) |  |
| strongly support | 83 (25.6%) |  |
| Support | 102 (31.5%) |  |
| Neutral | 89 (27.5%) |  |
| Weight-management apps record user’s data accurately, n (%). (N=324) | |  |
| Strongly disagree | 5 (1.5%) |  |
| Disagree | 34 (10.5%) |  |
| strongly support | 59 (18.2%) |  |
| Support | 99 (30.6%) |  |
| Neutral | 114 (35.2%) |  |
| I don't use apps that record my health information | 6 (1.9%) |  |
| App purchases n (%). (N=324) | |  |
| I wouldn't pay anything | 180 (55.6%) |  |
| SR15 or less | 50 (15.4%) |  |
| SR16 to SR22 | 43 (13.2%) |  |
| More than SR23 | 46 (14.2%) |  |
| Reasons for discontinuing use n (%). (N= 195) | |  |
| loss of interest | 64 (32.8%) |  |
| hidden costs | 53 (27.1%) |  |
| monitoring by a specialist was not offered | 27 (13.8%) |  |
| difficulty of using the app | 21 (10.7%) |  |
| language barrier | 18 (9.2%) |  |
