# Supplementary material for: Weight Management Apps in Saudi Arabia: Evaluation of Features and Quality
Source: JMIR Mhealth Uhealth. 2020 Oct 26;8(10):e19844. doi: 10.2196/19844 (PMC7652688; doi:10.2196/19844)
Supplement: Multimedia Appendix 4 [file mhealth_v8i10e19844_app4.docx]

**Multimedia Appendix 4. Apps that participants used for weight-management, n=267**

| **App name** | **n (%)** |
| --- | --- |
| MyFitnessPal | 145 (54.3%) |
| Health app | 16 (6.0%) |
| StepsApp Pedometer | 13 (4.9%) |
| Fitbit: Health & Fitness | 10 (3.8%) |
| Soarrate | 10 (3.8%) |
| Lose it calorie counter | 9 (3.4%) |
| Lifesum-Diet & food Diary | 8 (3.0%) |
| Adaad alsoaraat | 8 (3.0%) |
| mDiet | 6 (2.2%) |
| Calorie counter by fat secret | 5 (1.9%) |
| Rashaqa adad alsoarat | 4 (1.5%) |
| Gymnadz | 3 (1.1%) |
| Cronometer. Nutrition Tracker | 3 (1.1%) |
| Loss weight in 30 days | 3 (1.1%) |
| Zero | 3 (1.1%) |
| Hasebat alsoraat | 3 (1.1%) |
| Weight Tracker | 2 (0.7%) |
| Diet organizer | 2 (0.7%) |
| Nutrition | 2 (0.7%) |
| Pacer Pedometer | 2 (0.7%) |
| Nike | 2 (0.7%) |
| My diet coach-weight loss | 2 (0.7%) |
| Better me | 2 (0.7%) |
| My plate calorie | 2 (0.7%) |
| Alwazan almethali | 2 (0.7%) |
|  |  |
