# Supplementary material for: Weight Management Apps in Saudi Arabia: Evaluation of Features and Quality
Source: JMIR Mhealth Uhealth. 2020 Oct 26;8(10):e19844. doi: 10.2196/19844 (PMC7652688; doi:10.2196/19844)
Supplement: Multimedia Appendix 5 [file mhealth_v8i10e19844_app5.docx]

**Multimedia Appendix 5. Weight-management apps and their ratings in the app store**

| **Apps name** | **App language** | **App rating in app store** |
| --- | --- | --- |
| Lose weight for men¹ | Arabic & English | 5.0 |
| Pacer Pedometer¹ | English | 4.8 |
| 7min workout fitness app | English | 4.8 |
| MyFitnessPal¹ | English | 4.8 |
| Lifesum-Diet & food Diary¹ | English | 4.7 |
| StepsApp Pedometer¹ | Arabic & English | 4.7 |
| Lose it calorie counter¹ | Arabic & English | 4.7 |
| Calorie counter by fat secret¹ | English | 4.7 |
| Weight tracker¹ | Arabic & English | 4.6 |
| Rashaqa adad alsoara | Arabic | 4.6 |
| My diet Coach-weight loss¹ | English | 4.4 |
| Diet | Arabic | 4.1 |
| Tmarin manzliah | Arabic | 4.1 |
| Fitbit: Health & Fitness¹ | English | 4.1 |
| Hesab alwazan almethali | Arabic | 4.0 |
| FUDC¹ | Arabic | 3.8 |
| Adaad alsoaraat | Arabic | 3.6 |
| Rajeem 7kilo fi esboaa | Arabic | 3.3 |
| Soarrate | Arabic | 3.1 |
| Alwazan almethali | Arabic | 3.1 |
| mDiet¹ | Arabic | 2.9 |
| Rajem sareea | Arabic | 2.8 |
| Monabeh alsoaraat¹ | Arabic | 2.1 |
| ¹ In app purchase |  |  |
