# Supplementary material for: Weight Management Apps in Saudi Arabia: Evaluation of Features and Quality
Source: JMIR Mhealth Uhealth. 2020 Oct 26;8(10):e19844. doi: 10.2196/19844 (PMC7652688; doi:10.2196/19844)
Supplement: Multimedia Appendix 6 [file mhealth_v8i10e19844_app6.docx]

**Multimedia Appendix 6. The Mobile App Rating Scale mean scores for weight-management apps stratified by app language**

| App language | No. of apps | Mean Engagement | Mean  Functionality | Mean  Aesthetics | Mean  information | Mean  MARS | Mean  Subjective |
| --- | --- | --- | --- | --- | --- | --- | --- |
| Arabic | 12 | 2.9 | 3.3 | 2.7 | 2.4 | 2.8 | 1.8 |
| Arabic and English | 4 | 4.0 | 4.4 | 3.8 | 3.4 | 3.9 | 3.2 |
| English | 7 | 4.2 | 3.8 | 3.9 | 3.6 | 3.9 | 3.3 |
