# Supplementary material for: Weight Management Apps in Saudi Arabia: Evaluation of Features and Quality
Source: JMIR Mhealth Uhealth. 2020 Oct 26;8(10):e19844. doi: 10.2196/19844 (PMC7652688; doi:10.2196/19844)
Supplement: Multimedia Appendix 7 [file mhealth_v8i10e19844_app7.docx]

**Multimedia Appendix 7. The Mobile App Rating Scale mean scores and the number of users who reported using the apps**

| Weight-management app | MARS  Mean score | Survey  n (%) |
| --- | --- | --- |
| Lose weight for men | 4.4 | 0 (0.0 %) |
| Rashaqa adad alsoarat | 4.4 | 4 (1.5%) |
| MyFitnessPal | 4.3 | 145 (54.3%) |
| Fitbit: Health & Fitness | 4.3 | 10 (3.8%) |
| StepsApp Pedometer | 4.2 | 13 (4.9%) |
| Lose it calorie counter | 4.0 | 9 (3.4%) |
| Calorie counter by fat secret | 4.0 | 5 (1.9%) |
| Pacer Pedometer | 3.9 | 2 (0.7%) |
| Lifesum-Diet & food Diary | 3.9 | 8 (3.0%) |
| 7min workout fitness app | 3.9 | 0 (0.0%) |
| FUDC | 3.6 | 0 (0.0%) |
| mDiet | 3.3 | 6 (2.2%) |
| Adaad alsoaraat | 3.3 | 8 (3.0%) |
| Soraate | 3.3 | 10 (3.8%) |
| Weight Tracker | 3.0 | 2 (0.7%) |
| My diet Coach-weight loss | 3.0 | 2 (0.7%) |
| Tmarin manzliah | 2.9 | 0 (0.0%) |
| Alwazan almethali | 2.8 | 2 (0.7%) |
| Hesab alwazan almethali | 2.6 | 0 (0.0%) |
| Monabeh alsoaraat | 2.3 | 0 (0.0%) |
| Diet | 2.2 | 0 (0.0%) |
| Rajeem 7kilo fi esboaa | 1.7 | 0 (0.0%) |
| Rajem sareea | 1.7 | 0 (0.0%) |
|  |  |  |
