# Supplementary material for: Weight Management Apps in Saudi Arabia: Evaluation of Features and Quality
Source: JMIR Mhealth Uhealth. 2020 Oct 26;8(10):e19844. doi: 10.2196/19844 (PMC7652688; doi:10.2196/19844)
Supplement: Multimedia Appendix 8 [file mhealth_v8i10e19844_app8.docx]

**Multimedia Appendix 8. Evidence-based Strategy Assessment scores and the number of users who reported using the apps**

| Weight-management app | EBSs  n (%) | Survey  n (%) |
| --- | --- | --- |
| MyFitnessPal | 7 (87.5%) | 145 (54.3%) |
| Fitbit: Health & fitness | 7 (87.5%) | 10 (3.8%) |
| Rashaqa adad alsoarat | 6 (75.0%) | 4 (1.5%) |
| FUDC | 5 (62.5%) | 0 (0.0%) |
| Pacer Pedometer | 5 (62.5%) | 2 (0.7%) |
| Calorie counter by fat secret | 5 (62.5%) | 5 (1.9%) |
| Adaad alsoaraat | 5 (62.5%) | 8 (3.0%) |
| Soarrate | 5 (62.5%) | 10 (3.8%) |
| Weight Tracker | 4 (50.0%) | 2 (0.7%) |
| Lose it calorie counter | 4 (50.0%) | 9 (3.4%) |
| My diet Coach-weight loss | 3 (37.5%) | 2 (0.7%) |
| mDiet | 3 (37.5%) | 6 (2.2%) |
| Lifesum-Diet & food Diary | 3 (37.5%) | 8 (3.0%) |
| Tmarin manzliah | 3 (37.5%) | 0 (0.0%) |
| 7min workout fitness app | 3 (37.5%) | 0 (0.0%) |
| Lose weight for men | 3 (37.5%) | 0 (0.0%) |
| StepsApp Pedometer | 3 (37.5%) | 13 (4.9%) |
| Alwazan almethali | 2 (25.0%) | 2 (0.7%) |
| Diet | 2 (25.0%) | 0 (0.0%) |
| Hesab alwazan almethali | 2 (25.0%) | 0 (0.0%) |
| Monabeh alsoaraat | 1 (12.5%) | 0 (0.0%) |
| Rajeem 7kilo fi esboaa | 1 (12.5%) | 0 (0.0%) |
| Rajem sareea | 1 (12.5%) | 0 (0.0%) |
